# UDP-α-D-glucose 6-dehydrogenase: a promising target for glioblastoma

**DOI:** 10.18632/oncotarget.26670

**Published:** 2019-02-22

**Authors:** C. Rory Goodwin, A. Karim Ahmed, Shuli Xia

**Affiliations:** Department of Neurology, Hugo W. Moser Research Institute at Kennedy Krieger, Johns Hopkins School of Medicine, Baltimore, MD, USA

**Keywords:** glioblastoma, UGDH, extracellular matrix, DNA methylation, KLF4

Grade IV glioma, glioblastoma (GBM), is characterized by diffuse infiltration through brain parenchyma and invasion into the adjacent brain structures. Extracellular Matrix (ECM) is an important component of the tumor microenvironment; and a disrupted ECM is essential for GBM infiltration and invasion. The infiltrative phenotype contributes to GBM resistance to surgical removal and chemo/radiotherapy [1], therefore, it is critical to understand the molecular modulators of tumor cell migration and invasion. Krüppel-like factor 4 (KLF4), one of the Yamanaka factors, plays a vital role in cell migration, survival, the pluripotency of stem cells, and maintenance of cancer stem cells [2]. This epithelially enriched, zinc finger-containing transcription factor has been reported to function as a sequence-specific methylated CpG (mCpG) reader and activate transcription [3]. The KLF4-mCpG interaction also promotes GBM migration/invasion [4]; however, downstream factors of KLF4-mCpG binding activity involved in cell migration have been poorly understood.

In our prior study [4], we sought to characterize the gene regulatory elements associated with KLF4 function in human GBM cells. A site-specific mutant of KLF4 was generated and compared with KLF4 wild type (WT). KLF4 R458A lacks the ability to bind to mCpG but retains KLF4 binding to canonical non-mCpG containing DNA sequence [3]. Interestingly, KFL4 WT expression in GBM cells resulted in significantly increased cell adhesion and migration; whereas KLF4 R458A expression had no impact. Because the only difference between the cell lines was the ability of KLF4 to bind to methylated DNA, the findings suggested the increased cell adhesion and migration occurred *via* mCpG-dependent KLF4 activity. To identify the transcriptional network regulated by KLF4-mCpG interactions, whole genome RNA-sequencing (RNA-seq) was performed, demonstrating significant transcriptional regulation of 613 genes—among which 82% (*N* = 500) were affected by KLF4 WT expression only. Among the 500 genes, 62% (*N* = 308) had upregulated gene expression, suggesting they were activated by KLF4 *via* a methylation-dependent mechanism. This was validated by comparing gene expression in cells treated with 5-aza-2′-deoxycytidine (5-Aza), a DNA methytransferase inhibitor. To identify KLF4-mCpG direct target genes, whole genome chromatin immunoprecipitation-sequencing (ChIP-seq) was performed and overlapped with the RNA-seq datasets. The study found 116 target genes directly activated by mCpG-dependent KLF4 binding, with the majority (72%) of these genes associated with highly methylated *cis*-regulatory elements, as revealed by the whole genome bisulfite sequencing. In addition to transcriptional transactivation, ChIP-seq analysis of one activating (H3K27ac) and two repressive (H3K9me3 and H3K27me3) histone marks demonstrated that KLF4 WT binding to mCpGs triggered histone modification and chromatin remodeling.

**Figure 1:**
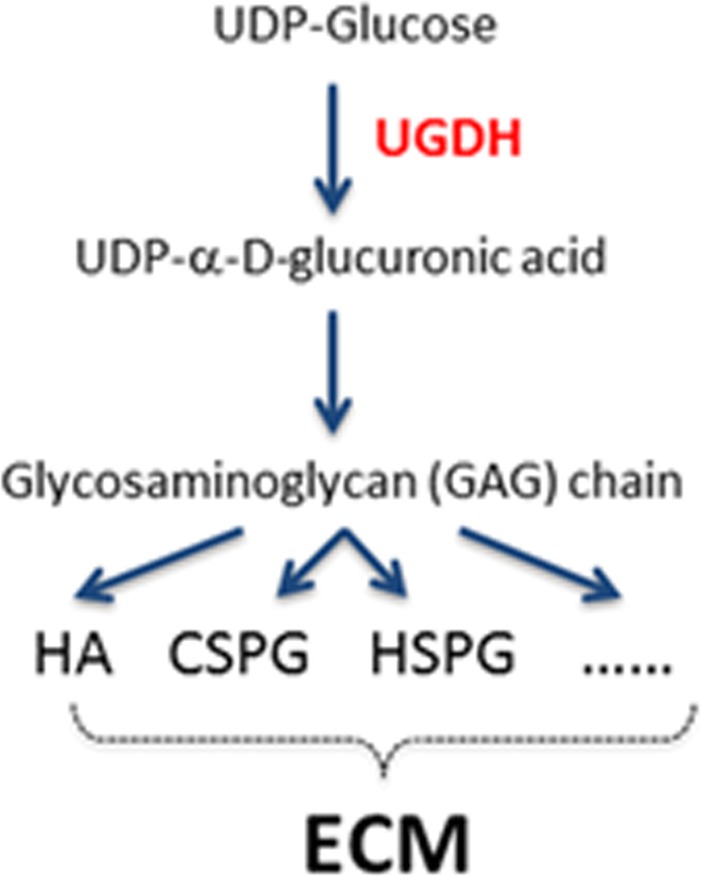
UGDH is the rate-limiting enzyme to synthesize ECM HA: hyaluronic acid; CSPG: chondroitin sulfate proteoglycan; HSPG: heparan sulfate proteoglycans

The top 20 genes upregulated by KLF4-mCpG interactions, associated with migration and cytoskeletal reorganization, were selected for detailed functional analysis. Among them, cytosolic enzyme UDP-glucose 6-dehydrogenase (UGDH) is responsible for the production of UDP-α-D-glucuronic acid [5], an essential precursor of glycosaminoglycan (GAG) and proteoglycan in the ECM (Figure 1). Recent evidence has demonstrated the importance of GAG production and the role of the ECM in the progression and invasion of GBM, among other solid tumors [6, 7]. Notably, GAGs are essential for cell proliferation, survival, and differentiation. From human samples, UGDH was upregulated in GBM samples when compared with normal brain specimens; and increased UGDH expression was associated with poor survival in a subset of GBMs. As such, the aim of the study by Oyinlade et al [8] was to determine whether targeting UGDH, a downstream target of KLF4-mCpG and a rate-limiting enzyme in the synthesis of ECM components, affected cellular phenotypes associated with GBM malignancy [4]. First, using ChIP-PCR, the authors demonstrated preferential binding of KLF4 WT, but not KLF4 R458A, to *cis*-regulatory regions of the UGDH gene. To determine whether UGDH expression was upregulated *via* a methylation-dependent mechanism, 5-Aza was administered and KLF4 binding to the *cis*-regulatory regions of UGDH was assessed. The study found a complete loss of KLF4 WT binding to UGDH *cis*-regulatory regions in the presence of 5-Aza, supporting the hypothesis that UGDH transcriptional activation was under the control of KLF4-mCpG interactions.

Two cellular systems, human GBM U87 cells and GBM neurosphere cells enriched for tumor-initiating cancer stem cells, were utilized to assess the impact of UGDH loss-of-function. Lentiviral transduction with UGDH shRNA dramatically knocked down UGDH expression and resulted in a marked reduction in GAGs, cell migration, and cell motility in both cell lines. Interestingly, hyaluronic acid supplementation (a key cellular GAG) rescued in migration inhibition in UGDH knockdown cells, suggesting that GAG reduction directly impacted migration. UGDH knockdown also resulted in decreased cell proliferation and clonogenicity—illustrated by a decreased ability to form colonies and neurospheres in GBM neurosphere models. Decreased UGDH expression caused a reduction in cyclin E and cyclin D1, leading to delayed G1/G0 to S-phase cell cycle transition. Most importantly, *in vivo* studies with intracranial xenografts demonstrated that UGDH knockdown reduced tumor growth, migration, and the expression of critical ECM components (i.e. tenascin C and brevican).

These studies highlight the importance of epigenetic mechanisms, i. e. DNA methylation, in gene activation and GBM malignancy including migration and proliferation. The work from Oyinlade reveals the UGDH enzyme is critical for ECM construction, which in turn affects the invasive and proliferative phenotypes of GBM. Future studies should be aimed at evaluating the anti-tumor potential of targeting UGDH in immunocompetent mouse models, either alone or combined with other therapeutic strategies. Furthermore, additional studies should seek to address whether these findings can be translated to other cancer models such as brain and/or bone metastases.
